# Genome-wide identification of ATP binding cassette (ABC) transporter and heavy metal associated (HMA) gene families in flax (*Linum usitatissimum* L.)

**DOI:** 10.1186/s12864-020-07121-9

**Published:** 2020-10-19

**Authors:** Nadeem Khan, Frank M. You, Raju Datla, Sridhar Ravichandran, Bosen Jia, Sylvie Cloutier

**Affiliations:** 1 Ottawa Research and Development Center, Agriculture and Agri-Food Canada, 960 Carling Avenue, Ottawa, ON K1A 0C6 Canada; 2 Department of Biology, University of Ottawa, 30 Marie Curie, Ottawa, ON K1N 6N5 Canada; 3 Aquatic and Crop Resource Development, National Research Council Canada, 110 Gymnasium Place, Saskatoon, SK S7N 0W9 Canada

**Keywords:** Flax, ABC transporter, HMA, Gene duplication, Expression profiling

## Abstract

**Background:**

The recent release of the reference genome sequence assembly of flax, a self-pollinated crop with 15 chromosome pairs, into chromosome-scale pseudomolecules enables the characterization of gene families. The ABC transporter and HMA gene families are important in the control of cadmium (Cd) accumulation in crops. To date, the genome-wide analysis of these two gene families has been successfully conducted in some plant species, but no systematic evolutionary analysis is available for the flax genome.

**Results:**

Here we describe the ABC transporter and HMA gene families in flax to provide a comprehensive overview of its evolution and some support towards the functional annotation of its members. The 198 ABC transporter and 12 HMA genes identified in the flax genome were classified into eight ABC transporter and four HMA subfamilies based on their phylogenetic analysis and domains’ composition. Nine of these genes, i.e., *LuABCC9, LuABCC10*, *LuABCG58, LuABCG59*, *LuABCG71*, *LuABCG72*, *LuABCG73, LuHMA3*, and *LuHMA4*, were orthologous with the Cd associated genes in *Arabidopsis*, rice and maize. Ten motifs were identified from all ABC transporter and HMA genes. Also, several motifs were conserved among genes of similar length, but each subfamily each had their own motif structures. Both the ABC transporter and HMA gene families were highly conserved among subfamilies of flax and with those of *Arabidopsis*. While four types of gene duplication were observed at different frequencies, whole-genome or segmental duplications were the most frequent with 162 genes, followed by 29 dispersed, 14 tandem and 4 proximal duplications, suggesting that segmental duplications contributed the most to the expansion of both gene families in flax. The rates of non-synonymous to synonymous (*Ka/Ks*) mutations of paired duplicated genes were for the most part lower than one, indicative of a predominant purifying selection. Only five pairs of genes clearly exhibited positive selection with a *Ka/Ks* ratio greater than one. Gene ontology analyses suggested that most flax ABC transporter and HMA genes had a role in ATP binding, transport, catalytic activity, ATPase activity, and metal ion binding. The RNA-Seq analysis of eight different organs demonstrated diversified expression profiling patterns of the genes and revealed their functional or sub-functional conservation and neo-functionalization.

**Conclusion:**

Characterization of the ABC transporter and HMA gene families will help in the functional analysis of candidate genes in flax and other crop species.

## Background

ATP binding cassette (ABC) transporter genes are ubiquitous across the three domains of life: Eukarya, Eubacteria and Archaea [1, 2]. Plant genomes harbor more than 100 ABC transporters which are involved in a broad range of biological functions [3]. ABC transporters comprise at least four domains: two transmembrane domains (TMDs) embedded in the membrane bilayer, and two nucleotide-binding domains (NBDs) located in the cytoplasm [1]. The structure of the TMDs is highly diverse and varies in the number of transmembrane helices, whereas the NBDs have highly conserved helices [4]. The ABC transporters are further categorized into full-size transporters with two NBDs and two TMDs and half-size transporters with only one of each domain [5]. Therefore, two half-size transporters must form either a homodimer or a heterodimer to be functionally active.

In plant genomes, ABC transporters are categorized into eight different subfamilies (ABCA-ABCG and ABCI) [3]. Proteins belonging to the ABCA-ABCD subfamilies have a forward domain organization (TMD-NBD) whereas ABCG and ABCI subfamilies have an inverse domain organization (NBD-TMD) [6]. ABCE and ABCF possess only two NBDs and are designated as soluble proteins [6]. In *Arabidopsis*, 130 ABC transporter genes have been identified but few have been functionally characterized [7]. Previous studies have shown that ABC transporters participate in a wide range of processes including the transport of ions, carbohydrates, lipids, xenobiotics, antibiotics, drugs, and heavy metals [8–10]. The two members of the ABCB gene family in *Arabidopsis* (*AtABCB1* and *AtABCB2*) are auxin transporters and the overexpression of *AtABCB1* causes the elongation of hypocotyl cells [11, 12]. Several members of the ABCC subfamily are responsible for phytate transport as exemplified in *Arabidopsis* (*AtABCC5*) [13], maize (*ZmABCC4*) [14] and rice (*OsABCC13)* [15]. Two other ABCC transporters (*AtABCC1* and *AtABCC2*) mediate tolerance to both cadmium (Cd) and mercury by vacuolar sequestration [16]. The ABCF subfamily member *AtABCF3* in *Arabidopsis* is involved in root growth and development [17]. ABCG subfamily members were reported to be involved in cuticle formation and Cd tolerance such as in *Arabidopsis* (*AtABCG32*) [18] and rice (*OsABCG31* and *OsABCG36*) [19, 20]. Also, the ABC transporter *AtABCG36* in *Arabidopsis* was shown to mediate Cd uptake in the epidermal cells of roots [21] and to be up-regulated by a Cd treatment [22].

Heavy metal (HM) pollution in food, water, and soil is hazardous to human health. HMs have become one of the major concerns across the globe due to the extensive industrialization and because of their direct and indirect effects on soil and crop productivity [23]. HMs such as zinc, copper, manganese, cobalt, and nickel are essential for various biological processes [24]. In contrast, other HMs such as arsenic, lead, and Cd are highly toxic to plants and negatively affect crop productivity [23]. Many HMA genes have been shown to play specific functions in different plant species. For example, *OsHMA2* is associated with vascular tissue loading of zinc and tonoplast localization in rice [25]. *OsHMA3,* localized in the tonoplasts, translocates Cd to the roots while *OsHMA4* transports copper to the roots [26]. *HvHMA1* is involved in zinc and Cd translocation into barley grain [27]. In wheat, HMA genes also play an important role in Cd translocation and are localized in the plasma membrane [28]. Overexpression of *AtHMA3* in *Arabidopsis* resulted in a 2- to 3-fold increase in Cd accumulation when compared to wild-type plants [29]. These cited studies provide an overview of the importance of both ABC transporter and HMA genes in various plant species, but no systemic studies have been reported in flax.

The initial draft of the flax genome sequence was produced using whole-genome shotgun (WGS) sequencing with short reads obtained on the Illumina sequencing platform [30]. A de novo assembly generated 88,384 scaffolds, totaling 318 Mb and representing ~ 81% of the estimated ~ 370 Mb flax reference genome [31]. Thus, the availability of this recent update of the flax genome (version 2.0) constitutes a genomic resource that allows the identification of gene families, evolutionary relationships, and structural analyses. To date, ABC transporter and HMA gene families have been studied in several plant species including *Oryza sativa* and *Arabidopsis thaliana* [32], *Zea mays* [33, 34], *Brassica rapa* [7], *Brassica napus* [35], *Triticum aestivum* [36], and *Vitis vinifera* [37]. A previous report on ABC transporters in flax has been published [38], but classification patterns, gene duplications, evolutionary and collinear relationships, structural and correlation analyses of paralogous pairs, and the identification of the Cd-associated genes covered herein were not explored in this prior publication. Several other gene families have also been identified in flax, based on bioinformatics analysis such as aquaporin [39], dirigent [40], chalcone [41], detoxification efflux carriers [42], and a ubiquitous glycosyltransferase [43]. In this research work, we hypothesized that either whole genome duplications (WGDs) or tandem events contributed to the expansion of the ABC transporter and HMA gene families in flax. Therefore, we studied the phylogenetic relationships, gene annotation, physicochemical properties, chromosomal distribution, gene synteny, protein-protein interactions (PPIs), and gene duplications of all predicted ABC transporter and HMA genes of the flax genome to understand their evolution and hypothesize their putative functions. Finally, we examined the gene ontology (GO) and expression profiling of ABC transporter and HMA genes in eight tissues, namely root, seed, ovary, and embryo at five different stages (heart, globular, torpedo, mature and cotyledon). This comprehensive analysis is the first report on ABC transporter and HMA genes in flax, providing gene candidate information for future marker association studies on heavy metal accumulation including Cd.

## Results

### ABC transporter and HMA genes in flax and their physicochemical properties

A total of 198 ABC transporter and 12 HMA genes were identified in the flax genome reference sequence of CDC Bethune [31]. The ABC transporter and the HMA genes were classified into eight and four subfamilies, respectively. These genes were denoted as *LuABCA1-LuABCA8*, *LuABCB1-LuABCB48*, *LuABCC1-LuABCC19*, *LuABCD1-LuABCD5*, *LuABCE1-LuABCE2*, *LuABCF1-LuABCF9*, *LuABCG1-LuABCG85*, *LuABCI1-LuABCI22*, and *HMA1-HMA12*. The basic information on these genes based on their subfamilies, including the protein identifier, coding sequence (CDS) length (bp), and protein properties such as the number of amino acid (aa) residues, molecular weight (kDa), isoelectric point (pIs), and grand average of hydropathicity (GRAVY), is listed in Table S1. The CDS length ranged from 663 to 7313 bp. The proteins had 220 to 2438 aa and a molecular weight of 25.54 to 273.69 kDa. The pIs ranged from 4.93 to 11.67 while the GRAVY values varied from − 0.606 to 0.619. Most genes (132/210) had positive GRAVY values, indicating hydrophobic properties.

LuABC and LuHMA proteins were predicted to localize to many subcellular compartments such as the plasma membrane, vacuole, endoplasmic reticulum, nucleus, cytoplasm, chloroplast, Golgi apparatus and mitochondrion. Further, several studies have also confirmed almost an identical localization of ABC transporter and HMA genes [7, 44].

### Annotation and phylogenetic analysis of ABC transporter and HMA genes in plant species

The gene annotation analysis of LuABC and LuHMA provides putative function(s) for each gene products (Table S2). In brief, based on the predicted function(s), most of the LuABC and LuHMA genes confirmed the presence of ABC transporter, ATP binding, and heavy metal ATPase domain functions. In addition, the LuABC subfamily were also involved in other important functions. For example, the subfamily LuABCC had multidrug resistance functions; the LuABCE subfamily encodes to RNAse l inhibitor protein, whereas members of the LuABCG subfamily were involved in regulating pleiotropic drug resistance. Other functions are also assumed to be assisted by LuABCs (Table S2). Thus, the annotation clearly shows the functional diversity of ABC transporters and HMAs in flax.

**Fig. 1:**
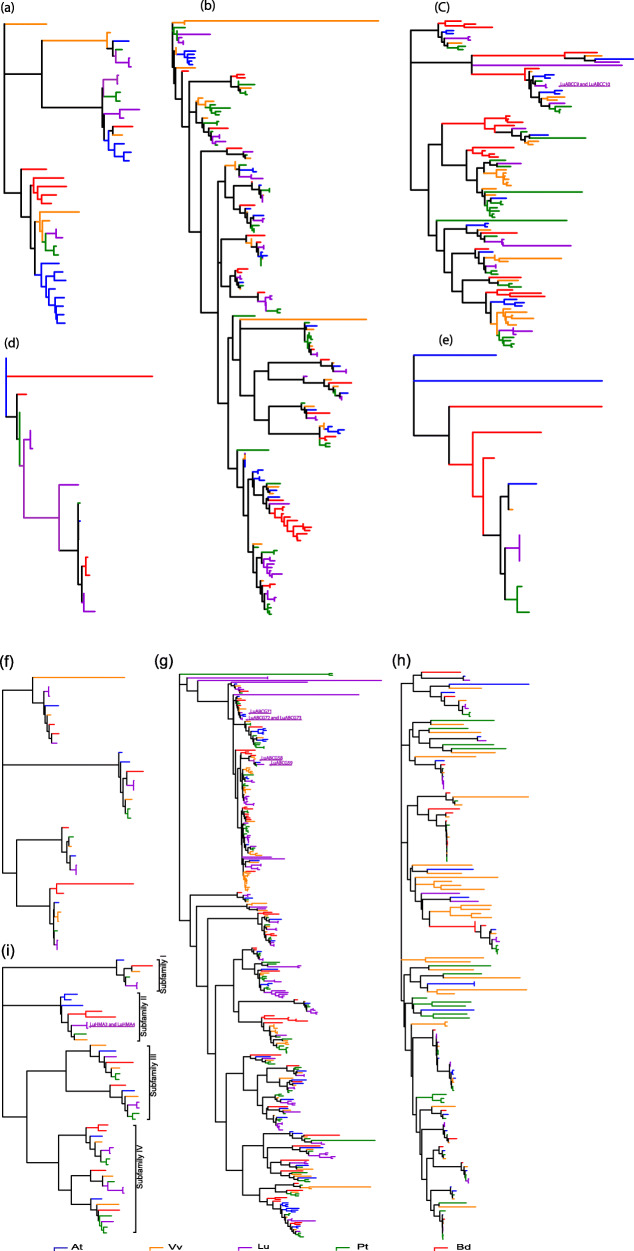
Phylogenetic relationships of eight subfamilies of the ABC transporter proteins (**a**-**h**) and four subfamilies of HMA proteins (**i**) in five species. *Arabidopsis thaliana* has 129 ABC transporter and 8 HMA proteins (AtABC and AtHMA), *Vitis vinifera* has 181 and 8 (VvABC and VvHMA), *Linum usitatissimum* has 198 and 12 (LuABC and LuHMA), *Populus trichocarpa* has 192 and 12 (PtABC and PtHMA), and *Brachypodium distachyon* has 133 and 9 (BdABC and LuHMA). The nine flax Cd candidate genes are indicated in green (**c**, **g** and **i**). Note: the taxa are available in supplementary Tables 3 and 4

Unrooted phylogenetic trees were constructed using the protein sequences for each of the eight ABC transporter and four HMA gene subfamilies of *Linum usitatissimum*, *Arabidopsis thaliana*, *Populus trichocarpa*, *Vitis vinifera*, and *Brachypodium distachyon* (Fig. 1 and Table S3). The phylogenetic relationships within LuABC, AtABC, PtABC, VvABC, and BdABC were highly conserved. Based on the phylogenetic relationships of flax and other species, the ABC transporter genes were divided into eight subfamilies: ABCA-ABCG and ABCI (Fig. 1a-h). Subfamilies ABCB and ABCG were the largest across all species, while ABCD and ABCE were the smallest based on the number of gene members per subfamily. With 81 members, ABCG was the largest subfamily and the dominant ABC transporter gene subfamily in flax.

The HMA genes of different species were, like flax, divided into four subfamilies based on their phylogenetic relationships (Fig. 1i and Table S4). Subfamily IV was the largest with 20 members, of which five belonged to flax. Subfamily I was the smallest with only six members across all the species studied. In short, the ABCG and HMA IV subfamilies had the highest number of genes in flax compared to other species except *Populus trichocarpa* in HMA. The distribution patterns of both ABC transporter and HMA genes and their subfamilies among five species are given in Table 1.

**Table 1:** The distribution patterns of ABC transporter and HMA genes in five plant species

Gene Families	Subfamilies	***Lu***	***At***	***Pt***	***Vv***	***Bd***
	ABCA	8	12	5	5	6
	ABCB	48	28	40	30	32
	ABCC	19	15	25	26	19
ABC transporter	ABCD	5	2	3	1	4
	ABCE	2	3	2	1	3
	ABCF	9	5	4	6	6
	ABCG	85	43	74	71	44
	ABCI	22	21	39	41	19
	Sub total	198	129	192	181	133
	I	2	1	1	1	1
	II	2	3	1	1	2
HMA	III	3	2	4	3	2
	IV	5	2	6	3	4
	Sub total	12	8	12	8	9
	Total	210	137	204	189	142

*Lu Linum usitatissimum*, *At Arabidopsis thaliana*, *Pt Populus trichocarpa*, *Vv Vitis vinifera*, and *Bd Brachypodium distachyon*

Based on previous reports on *Arabidopsis,* rice*,* and maize, several ABC transporter and HMA genes are associated with Cd tolerance, including *AtABCC1*, *AtABCC2*, *AtABCG36, AtHMA3* and *AtHMA4* in *Arabidopsis* [16, 21, 29, 45], *OsABCG31, OsABCG36* and *OsHMA2* in rice [20, 46], as well as *ZmHMA2* and *ZmHMA3* in maize [34]*.* We identified seven ABC transporter genes (*LuABCC9-LuABCC10*, *LuABCG58-LuABCG59,* and *LuABCG71-LuABCG73)*, and two HMA genes (*LuHMA3-LuHMA4*) which were orthologous with the above Cd-related genes (Fig. 1c, g and h). These genes are the most likely candidates for Cd accumulation in flax.

### Chromosomal localization, Syntenic relationships, and duplication of ABC transporter and HMA genes

**Fig. 2:**
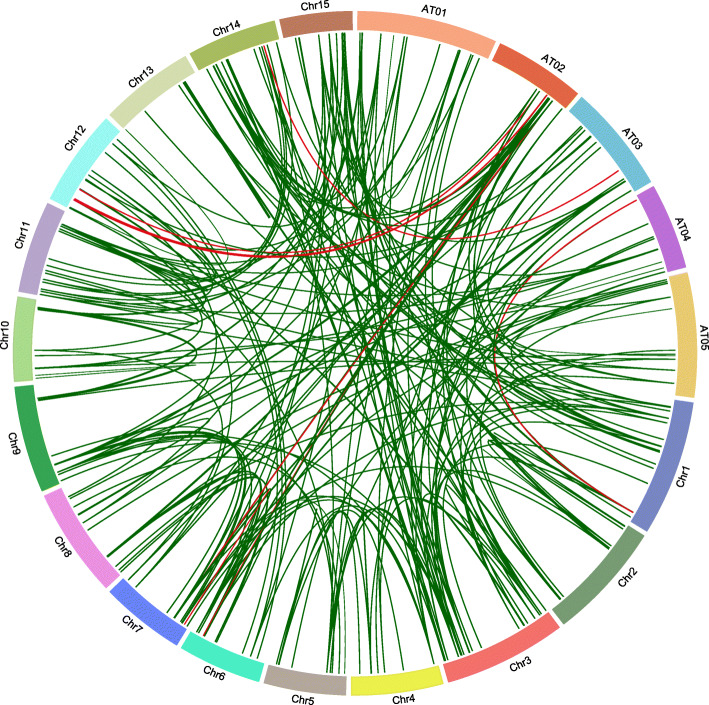
Chromosomal locations of the orthologous ABC transporter and HMA genes of flax and *Arabidopsis*. The 196 ABC transporter and 11 HMA genes on the 15 chromosomes of flax (Chr1–15) and the 129 AtABC transporter genes on the five chromosomes of *Arabidopsis* (AT01–05) are illustrated and orthologous relationships are indicated by green lines. The nine Cd associated genes are marked in red

A total of 196 LuABC and 11 LuHMA genes were located on the 15 chromosomes of flax and three of these genes (*LusABCG10*, *LusABCG15,* and *LuHMA5*) were found on scaffolds that have not been positioned onto any of the chromosomes (Table S1). LuABC gene subfamilies and genes per se are distributed unevenly across flax chromosomes. The largest number of ABC transporter and HMA genes was on Chr3 (23), followed by Chr11 (18), and Chr1 (17). The nine predicted Cd-accumulation candidate genes were scattered on multiple flax chromosomes: *LuABCG71* on Chr1*, LuABCG73* on Chr3, *LuABCG59* on Chr6*, LuABCC9* and *LuHMA3* on Chr7, *LuABCC10, LuABCG58* and *LuHMA4* on Chr12*,* and *LuABCG72* on Chr14 (Fig. 2). The gene collinearity analysis revealed high conservation among subfamilies of both ABC transporters and HMA with *Arabidopsis* orthologues (Fig. 2).

Four different types of gene duplications were observed from the identified ABC transporter and HMA genes, including 162 WGD (segmental), 29 dispersed, 14 tandem, and 4 proximal duplications. Only one ABC transporter gene (*LuABCI16*) was a singleton. Eight of the nine flax Cd candidate genes were of the segmental type and one (*LuHMA4*) was a tandem duplication. Thus, segmental duplications played a dominant role in the expansion of the ABC transporter and HMA gene families in flax and confirmed our hypothesis.

### Synonymous and non-synonymous substitution rates, gene structure analysis and motif composition

The synonymous (*Ks*) and non-synonymous (*Ka*) values were estimated based on the duplicated pairs of genes across the flax genome. The *Ka*/*Ks* ratios of five pairs (*LuABCG71/LuABCG72, LuABCG61/LuABCG64, LuABCG80/LuABCG69, LuABCG4/LuABCG3,* and *LuHMA6/LuHMA8)* exceeded one, suggesting positive selection. The remaining gene pairs underwent purifying selection with a *Ka/Ks* ratio of less than one. The estimated duplication time of LuABC and LuHMA gene pairs ranged from 1.53 to 28.27 million years ago (MYA), with an average of 8.59 MYA (Table S5).

**Fig. 3:**
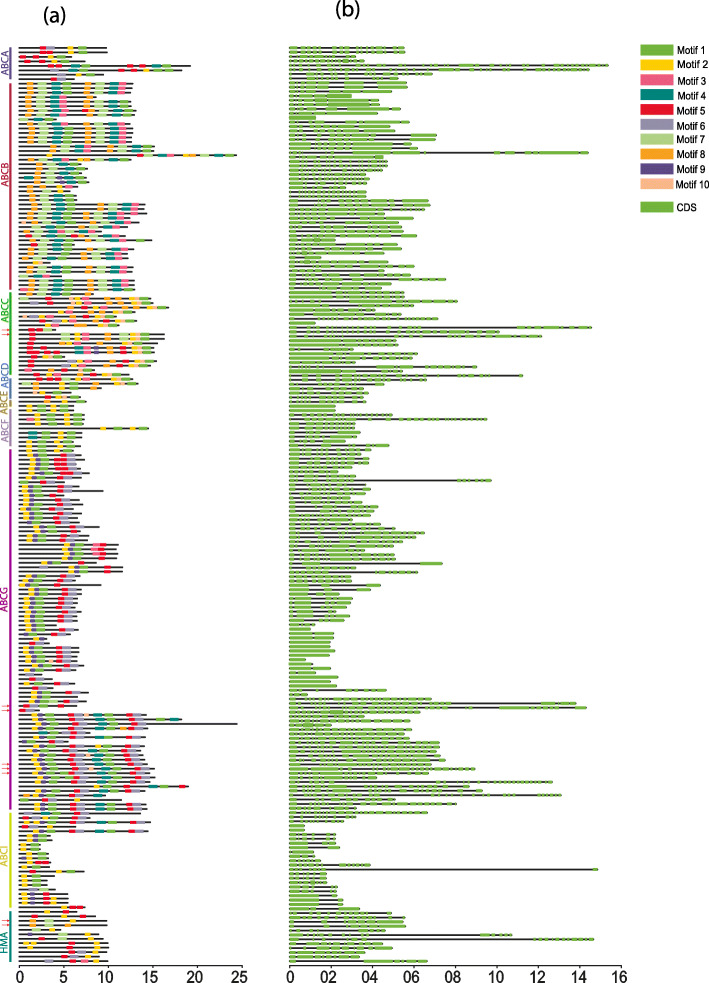
Motif structures (**a**) and gene structures (**b**) of subfamilies LuABCA-LuABCG, LuABCI and LuHMA in flax. The nine potential Cd candidate genes are marked with red arrows. Motif 1–10 are displayed in different colors. The gene structure of LuABCA-LuABCG and LuABCI are based on the coding sequences (CDS) presented in green

Conserved motifs and gene structure organization of LuABCs and LuHMAs were analyzed to better understand the global conservation and diversification of these two gene families. A total of ten distinct conserved motifs were identified. Several motifs were highly conserved; for instance, motifs 2 and 5 commonly occurred among subfamilies LuABCA-LuABCI members as well as in HMA proteins (Fig. 3a). Of the ten motifs, motif 6 was prevalent in both ABC transporter and HMA proteins except in ABCB, ABCD, and ABCF subfamilies. Of the nine flax Cd candidate genes, three (*LuABCG71, LuABCG72,* and *LuABCG73*) consistently exhibited 9–10 of these motifs and similar gene lengths. However, distinct motif compositions existed among most of the subfamilies.

The exon structure analysis based on the coding sequences of LuABCs and LuHMAs showed diversification between and within subfamilies. Specifically, 19 ABC transporter genes, including *LuABCC7*, *LuABCC9*–11, *LuABCD1*, *LuABCF4*, *LuABCG59–62*, *LuABCG70–71*, *LuABCG75*, *LuABCG78–81*, *LuABCG88,* and *LuABCG85*, contain a variable number of exons ranging from 20 to 31 (Fig. 3b). The number of exons was highly conserved within ABC transporter gene subfamilies. The large number of exons observed in *LuABCA6* (38) and *LuABCA5* (40) was unique because the remaining ABC transporters and HMA had 1–19 exon(s).

### Gene ontology (GO) and expression profiling

To predict the regulatory functions of the LuABC and LuHMA genes in flax, we performed gene ontology (GO) analyses. The GO terms were categorized into three subgroups: molecular function (MF), cellular component (CC), and biological process (BP), as described in Table S6. The LuABC and LuHMA proteins were enriched for MF such as ATP binding (GO:0005524), ATPase activity (GO:0016887), transporter activity (GO:0005215), catalytic activity (GO:0003824), kinase activity (GO:0016301), and metal ion binding (GO:0046872). CC GO terms associated with LuABC included the integral component of membrane (GO:0016021), intracellular (GO:0005622), membrane (GO:0016020), and integral component membrane (GO:0016021). BP terms comprised transport (GO:0006810), transmembrane transport (GO:0055085), signal transduction (GO:0007165), GTPase-mediated signal transduction (GO:0007264), and cation transport (GO:0006812). Taken together, GO terms indicated roles in central processes involving ATP binding and metal ion transport but also a wide range of other processes and activities in flax.

**Fig. 4:**
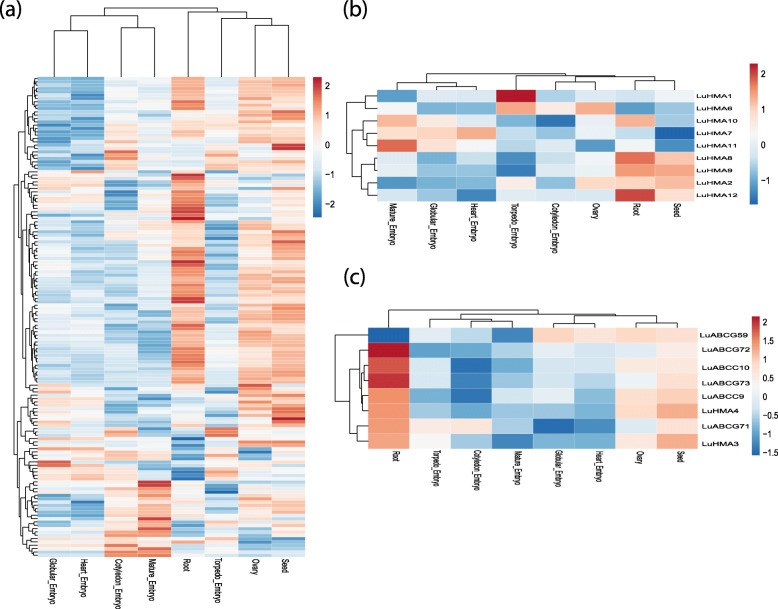
Expression profiling of the 160 differentially expressed genes in eight different tissues based on log fold-changes, including 143 LuABC genes (**a**), nine LuHMA genes (**b**), and eight potential Cd candidate genes (**c**). The red represents up-regulated genes and blue is for down-regulated ones. The remaining 48 LuABC and one each HMA (*LuHMA5*) and Cd (*LuABCG58*) genes were discarded because they were represented by less than 5 RPM (reads per million) after normalization of the data. Expression in anther tissue was used as a reference for expression analysis

The expression patterns of the LuABC and LuHMA genes in the root, seed, ovary, and five different stages of embryo development (heart, globular, torpedo, mature, and cotyledon) from RNA-Seq data are presented in a heatmap (Fig. 4). In general, the highest number of genes up-regulated for both LuABC and LuHMA genes was in seed (84/152), followed by root (75/152), and ovary (70/152) tissues. Among the five different embryo development stages, both LuABC and LuHMA genes showed a relatively weak expression considering that only 47, 41, 39, 37 and 36 of the 152 expressed genes were up-regulated in mature, cotyledon, torpedo, globular, and heart stages of embryo development, respectively. The remaining LuABC and LuHMA genes were not expressed or displayed a low-level expression in these different organs (Fig. 4a and b). The high expression level of LuABC and LuHMA genes in root and seed suggests that these genes might play a ubiquitous (housekeeping) transport role in these tissues in flax.

Eight of the nine flax Cd candidate genes were highly expressed in different tissues, including *LuABCG71, LuABCG72,* and *LuABCG73* in root and seed (Fig. 4c). Additionally, multidimensional scaling (MDS) also revealed differential gene expression between organs and high consistency of expression data between biological replicates (Figure S1).

### Functional evolution of duplicated genes and interaction network

Expression profiling can be mined to predict the functional fate of genes, and here, investigation of the mode and tempo of duplicated genes was performed to assess their functional evolution. We utilized and took advantage of RNA-Seq data to calculate the Pearson correlation coefficient (*r*) of the syntenic pairs across the eight different tissues used in our research. The significance of each expression level was tested based on the *r* values. Positive expression correlation was inferred when *r* values exceeded 0.156 (at a significance level of α = 0.05). Using this cut-off value, 42 of the 51 pairs had positive expression correlations, likely indicating functional conservation or sub-functionalization after duplication. The remaining nine pairs had correlation values below 0.068 or a negative correlation, suggesting putative neo-functionalization of at least one of the syntenic pairs (Table S7).

The protein-protein interaction networks of LuABC and LuHMA were examined and a dense network formed among the protein subfamilies (Figures S2a and b). However, a few of the ABC proteins did not interact: LuABCG6, LuABCG66, LuABCG68, LuABCG80, and LuABCG83 (Figure S2a). When the different subfamilies were compared, the LuABCG genes were preferentially retained in flax throughout evolution.

## Discussion

Here, we identified 198 ABC transporter and 12 HMA genes in flax, accounting for 0.484% of the total 43,384 annotated genes of its reference sequence [30]. The observed sequence divergence of LuABC and LuHMA genes and the variations in the physicochemical properties of their proteins are in line with the broad diversity of their biological functions. For example, based on the variations in GRAVY and pIs values among subfamilies of LuABC and LuHMA, we speculate that the members of these two gene families have the ability to respond to a variety of environmental cues at the micro- or macro-environment levels.

The domain composition analysis and phylogenetic tree validated the eight subfamilies LuABCA-LuABCG and LuABCI and the four subfamilies of LuHMA proteins. The results of the phylogenetic relationships between the genes were consistent with previous findings in *Arabidopsis thaliana, Brassica rapa,* and *Brassica napus* [7, 35]. Flax had more ABC transporter and HMA genes than any of the other five species investigated which included the dicots *Arabidopsis thaliana*, *Vitis vinifera*, *Populus trichocarpa*, and the monocot *Brachypodium distachyon*, despite having a smaller genome than all of them except *Arabidopsis*. In general, the LuABC and LuHMA genes were scattered on the phylogenetic tree, suggesting that the expansion of these gene families occurred before evolutionary divergence of the common ancestor.

Previous studies indicated that two ABCC, two ABCG, and two HMA genes in *Arabidopsis*, rice and maize were involved in Cd accumulation. The flax genes orthologous to these genes are potential candidates for Cd tolerance. Therefore, we identified nine flax Cd candidate genes, namely *LuABCC9–10*, *LuABCG58–59*, *LuABCG71–73*, and *LuHMA3–4*. We intend to validate these genes by genome-wide association study. However, the expression profiling of these genes provide us with possible functional roles. Several studies have demonstrated that ABC transporters and HMAs participate in various plants’ growth activities and stress tolerance. For instance, *HvABCG31* is essential for the retention of leaf water and *ABCB1* participates in auxin transport, and its overexpression regulates hypocotyl cell elongation [11, 12, 19]. Similarly, one member of ABCC and two ABCG i.e., *OsABCC1* and *AtBCG25* participate in arsenic accumulation and abscisic acid transport [47, 48], and its other member *AtABCG36* improves drought tolerance [49]. Further, *ABCG13* has been reported to be involved in petal elongation in *Arabidopsis* [50]. Metal transporters are known to play pivotal roles in numerous aspects of plants’ metabolism including essential and toxic metals’ distributions [51]. Thus, we hypothesized the possible functions of LuABC transporters and LuHMAs by examining their gene annotation, gene ontology, and gene expression in multiple tissues. Taken together, LuABCs and LuHMAs seem to play particular roles in ATP binding, transport and, metal ion binding activities. The gene expression results also revealed that the majority of the genes were highly expressed in one or more of the eight tissues evaluated, thereby confirming tissue-specific expression. Of the nine Cd gene candidates proposed, the four (*LuABCG71–73* and *LuABCC10*) up-regulated in root and seed tissues had a conserved gene structure, suggesting their putative redundant functions in these developmental tissues in flax.

Protein sequence analyses of gene families are needed to understand neo-functionalization and divergence [52]. Most angiosperms have undergone at least two WGD events [53] which are frequently associated with significant evolutionary switches that can contribute to the adaptability of species to a range of environments [54]. WGDs are associated with the development of distinct plant species, and gene duplications are a vital force in genomic evolution and functional divergence [55]. Similarly, in evolutionary history, most higher plants underwent polyploidization, a vital event in shaping plant genome [56]. Not surprisingly, flax has also undergone a palaeopolyploidization (23–44 MYA) and a mesopolyploidization (3.7–9 MYA) events [30, 31]. Our findings suggest an average estimated duplication divergence time of 8.59 MYA for LuABC and LuHMA genes, consistent with the most recent (3.7–9 MYA) WGD of the flax genome. Segmental and tandem duplications are the predominant mode of expansion in *Arabidopsis* [57]. In flax, four types of gene duplications were observed in the 210 ABC transporter and HMA genes. Segmental (WGD) duplications (77.14%) contributed by far the most to the expansion of the two gene families in flax, a common mode of expansion of gene families across various plant species [58–60]. The selection pressure analysis of duplicated gene pairs based on three categories (i.e., purifying, positive, and neutral selection) tends to provide valuable evolutionary information [61]. *Ka/Ks* ratio values of less than, equal to or greater than one signify purifying, neutral or positive selections, respectively [62, 63]. Most of the LuABC and LuHMA gene pairs underwent purifying selection. Our findings suggest that these pairs of genes largely contributed to growth and development, while the strong positive selection in a few genes is indicative of functional differentiation. Duplication of genes can lead to one of several fates such as functional or sub-functionalization, neo-functionalization and pseudogenization [62]. The correlation analysis based on the expression of syntenic pairs across tissues indicated only two fates: functional or sub-functionalization and neo-functionalization. Most genes pairs likely maintained the same function, thereby showing functional conservation. However, nine pairs exhibited neo-functionalization, indicating new function(s) for the two genes of the pairs. The structural diversity mainly contributed to the evolution of the gene families as indicated by evolutionary studies [64]. The observed diversity in a few LuABC and LuHMA genes may have been lost throughout evolution, and that might have contributed to their functional divergence after their loss or birth.

Taken together, these analyses suggest that the ABC transporter and HMA gene families in flax expanded over evolutionary time through gene duplication events. Among the nine putative Cd responsive genes, we observed consistencies of several properties for *LuABCG71* and *LUABCG72*. Both showed conserved gene structure with similar number of introns (20 and 16, respectively). The gene duplication analysis for this pair indicated a segmental duplication origin and both underwent positive selection. Their expression was up-regulated in root and seed tissues, and functional conservation was revealed through their Pearson correlation coefficient (PCC) (Figure S3). Also, the remaining four pairs exhibiting positive selection (i.e., *LuABCG61/LuABCG64, LuABCG80/LuABCG69, LuABCG4/LuABCG3*, and *LuHMA6/LuHMA8*) did not show as much consistency when compared with the two Cd genes based on their expression patterns and motif structures. In summary, the major intention of this study was to provide a comprehensive analysis of ABC and HMA genes which are associated with abiotic stress resistance, and more specifically resistance to Cd. Our intention was not only the identification of genes putatively involved in Cd accumulation in flax but also to understand their evolution. Further functional characterization is needed to validate the Cd-associated genes and to define which syntenic paralogous pairs underwent functional changes during evolution, either as a consequence of structural changes of the CDS or through expression profile changes. Both epigenetic and structural modifications in cis- or trans-elements have an impact on gene expression.

## Conclusion

A comprehensive sequence analysis of the ABC transporter and HMA gene families in flax was performed. We identified 198 LuABC and 12 LuHMA genes that clustered into eight ABC transporter (ABCA-ABCG and ABCI) and four HMA subfamilies. Among them, nine were predicted to be potentially involved Cd accumulation *in planta* based on homology with previously characterized genes in *Arabidopsis*, rice and maize. Their phylogenetic relationships, gene annotation, motif composition, gene structure, syntenic relationships, cis-regulatory elements, and gene ontology are reported. The gene duplication analysis suggested that four different types of duplications occurred among LuABC and LuHMA genes, namely WGD or segmental, tandem, dispersed, and proximal. WGD in flax contributed the most to the expansion of LuABC and LuHMA genes. The divergence estimates indicated that recent duplications (mesopolyploidization) occurred within these two gene families. The expression data illustrated the high degree of diversification, and the evolutionary fate of syntenic gene pairs, thereby showing their functional or sub-functional conservation and neo-functionalization. Our results provide insights into the evolution and divergence of LuABC and LuHMA genes in flax. These analyses will be foundational to future investigations into the biological functions of ABC transporter and HMA genes in flax, and will be especially helpful in conjunction with a marker association study for Cd accumulation in flax.

## Materials and methods

### Identification of ABC transporter and HMA genes, and their duplications

Two methods were used to identify ABC transporter genes in flax. Firstly, the ABC transporters were identified based on the 129 reference sequences [65] from the *Arabidopsis* genome (version 10.0, http://www.arabidopsis.org/) by BLASTP search method using an E-value cut-off of 1.0E-10 against the flax genome [30]. Secondly, we performed a Hidden Markov Model (HMM) search against the flax genome to confirm the presence of ABC transporter genes using HMMER (version 3.2.1) with the default options [66]. The ABC transporter domains included ABC transporter (PF00005), ABC-2 transporter (PF01061), ABC transporter transmembrane region (PF00664), cytochrome c polymerization (CYT) (PF01458) or mammalian cell entry (mce) related protein (PF02470). These domains were downloaded from the Pfam (version 32.0) database (http://pfam.xfam.org/) [67]. Similarly, the HMA genes were identified based on the eight reference gene sequences (Table S4) of *Arabidopsis* using BLASTP method as discussed above.

After merging the results, ABC transporter and HMA genes were further screened on the basis of their domain composition. The duplicated results between the two methods were eliminated. A total of 745 ABC transporter and HMA proteins were identified among all species other than those of *Arabidopsis thaliana*.

Sequences of the 15 chromosomes of flax (version 2.0) were obtained from NCBI under GenomeProject ID no. 68161 (accession numbers from CP027619–CP027633) [31] and protein sequences were downloaded from Phytozome (version 12.1) [68]. Genomic sequences of *Populus trichocarpa* (version 3.1), *Vitis vinifera* (version Genoscope12X), and *Brachypodium distachyon* (version 1.2) were obtained from Phytozome (http://phytozome.jgi.doe.gov/pz/portal.html) [68]. The obtained protein sequences of ABC transporter and HMA genes were further verified for ABC/HMA domain compositions using the NCBI-Conserved Domain database (http://www.ncbi.nlm.nih.gov/Structure/cdd/wrpsb.cgi) [69] and SMART (http://smart.embl-heidelberg.de/) [70]. Protein sequences with errors, short (< 100 aa), or without ABC or HMA domains were removed.

For the identification of flax Cd-associated genes, several ABC transporter and HMA responsive genes were used as a reference. These genes included *AtABCC1*, *AtABCC2*, *AtABCG36*, *AtHMA3* and *AtHMA4* in *Arabidopsis* [16, 21, 29, 45], *OsABCG31*, *OsABCG36* and *OsHMA2* in rice [20, 46], *ZmHMA2* and *ZmHMA3* in maize [34]. Nine Cd associated genes were identified by performing the BLASTP method using the same criteria as discussed above for ABC and HMA genes.

### Phylogenetic characterization of ABC transporter and HMA genes, and synonymous (*Ks*) and non-synonymous (*Ka*) substitution rates for duplicated genes

Multiple sequence alignments for ABC transporter or HMA protein sequences from *Linum usitatissimum*, *Arabidopsis thaliana*, *Populus trichocarpa*, *Vitis vinifera*, and *Brachypodium distachyon* were performed using MUSCLE (version 3.8.1551). Phylogenetic trees were then constructed using the MEGA (version 7.0) software [71] with the maximum likelihood (ML) method and the Jones, Taylor, and Thornton amino acid substitution model (JTT model). The JTT model was chosen based on the results using the option provided in MEGA to find the best fit model for evolution of ABC/HMA genes.

The different types of gene duplications in the flax genome were identified using MCScanX [72]. Synonymous (*Ks*) and non-synonymous substitution (*Ka*) rates were also calculated for duplicated gene pairs as previously described [58]. Also, a substitution rate of 1.5 × 10^− 8^ substitutions per synonymous site per year [73] was used to estimate the divergence time of duplicated genes.

### Conserved motifs, gene structure, and physicochemical parameters

Multiple Em for Motif Elicitation (version 5.1.0) [74] were used for scanning the conserved motifs of LuABC and LuHMA proteins. The maximum number of motifs of 10, with a minimum of 50 aa and a maximum of 100 aa, were set as parameters. TBtools (version 0.66) [75] was used to visualize both the motif composition and gene structure. The ExPASY PROTPARAM tool (http://web.expasy.org/protparam/) was accessed to determine the following physicochemical properties: molecular weight (MW), isoelectronic points (pI), and GRAVY values for each gene. The subcellular localization of both ABC and HMA genes was predicted using the web applications of WOLF PSORT (http://wolfpsort.hgc.jp/) [76] and BUSCA (http://busca.biocomp.unibo.it/) [77].

### Chromosomal location, gene Synteny analysis, and protein-protein interaction (PPI) analysis

The gene synteny between flax and *Arabidopsis* was analyzed based on the gene annotation data of both species and illustrated using shinyCircos [78]. The PPI analysis for all ABC transporter and HMA proteins was carried out using the online STRING server (version 11.0) (http://string-db.org/) [79] with the following parameters: medium score of 0.400, number of K means clustering of 3, and default values for the remaining options. The results of the interaction network were visualized using Cytoscape (version 3.4.0) [80].

### Plant materials, RNA sequencing and read data analysis

Flax cultivar CDC Bethune was planted in the greenhouse under growth conditions previously described [81]. Tissues from root, seed, anther, and ovary at five different embryonic developmental stages (heart, globular, torpedo, mature and cotyledon embryo) were collected for RNA extraction with two biological replicates for each tissue. Total RNA was extracted from each sample following the RNAqueous kit protocol (Ambion, Catalog #1912) and RNAqueous-Micro kit protocol (Ambion, Catalog #1931). The samples were homogenized in lysis buffer with polypropylene pestles in 1.5 ml Eppendorf tubes on ice. For RNA-Seq profile analysis, Illumina mRNA-Seq libraries were prepared using the TruSeq RNA kit (ver. 1, rev. A) according to the manufacturer’s instructions. An Agilent 2100 Bioanalyzer was used for quantification and quality assessment of sample libraries. For Illumina HiSeq 2000 sequencing, four indexed libraries were pooled per sequencing lane and 100bp paired-end sequencing was performed.

The raw reads were initially trimmed by trimmomatic [82] and the trimmed reads were aligned to the flax reference genome sequence using kallisto [30, 83]. The contigs with fewer than 5 reads per million (RPM) in at least one library were filtered out. Normalization was performed at trimmed mean of M-values (TMM) using edgeR [84]. A general linear model (GLM) was used with glmLRT function to identify differentially expressed genes with false discovery rate (FDR) less than 0.05 [84]. The anther results were used as a reference for expression analysis of all tissues. Thus, expression results were presented for the eight tissues by comparing them to those of the anther.

Heat maps were drawn based on normalized Log_2_ scale read counts using ClustVis [85]. Bidirectional cluster analysis was conducted using maximum distance and complete linkage method. Pearson correlation coefficient (*r*) based on expression data among the syntenic pairs of flax was calculated using Rstudio (version 3.6.0).

The GO analysis for ABC transporter and HMA genes in flax was conducted using the Phytozome database (http://phytozome.jgi.doe.gov/pz/portal.html) with keyword search options against the flax genome.

## Supplementary information

**Additional file 1: Figure S1.** Multidimensional scaling (MDS) plot of nine different tissues displaying the relative similarities between the biological replicates based on the log fold change values. **Figure S2.** Interaction network of ABC transporter genes (a) and HMA genes (b). **Figure S3.** Schematic representations of the two most stable genes among nine Cd candidate genes based on their conserved gene structure (a), gene expression (b), non-synonymous/synonymous substitution rates (c), and Pearson correlation coefficient (PCC) (d).

**Additional file 2: Table S1.** Basic information on the ABC transporter and HMA genes identified in flax.

**Additional file 3: Table S2.** Annotation information of LuABC and LuHMA genes in flax based on chromosomal position.

**Additional file 4: Table S3.** List of ABC transporters identified in different species.

**Additional file 5: Table S4.** List of HMA genes identified in different species.

**Additional file 6: Table S5.** Gene duplications of the syntenic gene pairs in flax.

**Additional file 7: Table S6.** Gene ontology (GO) of ABC transporter and HMA genes in flax.

**Additional file 8: Table S7.** Pearson correlation coefficients of syntenic gene pairs and their functional evolution.

## Data Availability

The RNA-Seq data of all the nine tissues have been deposited into the sequence read archive database with the link of https://www.ncbi.nlm.nih.gov/bioproject/PRJNA663265/, under the following accession numbers: SAMN16124659-SAMN16124667. The remaining data are available in the manuscript and its supplementary materials.

## Abbreviations

ABC: ATP Binding Cassette
HMA: Heavy Metal Associated
WGD: Whole Genome Duplication
GO: Gene Ontology
Cd: Cadmium
GLM: General Linear Model
FDR: False Discovery Rate
JTT: Jones, Taylor, and Thornton
ML: Maximum Likelihood
*Ks*: Synonymous
*Ka*: Non-synonymous
HMM: Hidden Markov Model
MDS: Multidimensional Scaling Plot
BCV: Biological Coefficient of Variation
RPM: Reads Per Million
TMDs: Transmembrane Domains
NBDs: Nucleotide-binding Domains
MYA: Million Years Ago

## References

[1] Rees DC, Johnson E, Lewinson O (2009). ABC transporters: the power to change. Nat Rev Mol Cell Biol.

[2] Jones PM, George AM (2004). The ABC transporter structure and mechanism: perspectives on recent research. Cell Mol Life Sci.

[3] Kang J, Park J, Choi H, Burla B, Kretzschmar T, Lee Y, Martinoia E (2011). Plant ABC transporters. Arabidopsis Book.

[4] Schneider E, Hunke S (1998). ATP-binding-cassette (ABC) transport systems: functional and structural aspects of the ATP-hydrolyzing subunits/domains. FEMS Microbiol Rev.

[5] Hollenstein K, Frei DC, Locher KP (2007). Structure of an ABC transporter in complex with its binding protein. Nature.

[6] Mishra AK, Choi J, Rabbee MF, Baek K-H (2019). In silico genome-wide analysis of the ATP-binding cassette transporter gene family in soybean (*Glycine max* L.) and their expression profiling. Biomed Res Int.

[7] Yan C, Duan W, Lyu S, Li Y, Hou X. Genome-wide identification, evolution, and expression analysis of the ATP-binding cassette transporter gene family in *Brassica rapa*. Front Plant Sci. 2017;8:349. 10.3389/fpls.2017.00349 PMC5355449 28367152

[8] Gadsby DC, Vergani P, Csanády L (2006). The ABC protein turned chloride channel whose failure causes cystic fibrosis. Nature.

[9] Pighin JA, Zheng H, Balakshin LJ, Goodman IP, Western TL, Jetter R, Kunst L, Samuels AL (2004). Plant cuticular lipid export requires an ABC transporter. Science.

[10] Sipos G, Kuchler K (2006). Fungal ATP-binding cassette (ABC) transporters in drug resistance & detoxification. Curr Drug Targets.

[11] Sidler M, Hassa P, Hasan S, Ringli C, Dudler R (1998). Involvement of an ABC transporter in a developmental pathway regulating hypocotyl cell elongation in the light. Plant Cell.

[12] Noh B, Murphy AS, Spalding EP (2001). Multidrug resistance-like genes of *Arabidopsis* required for auxin transport and auxin-mediated development. Plant Cell.

[13] Nagy R, Grob H, Weder B, Green P, Klein M, Frelet-Barrand A, Schjoerring JK, Brearley C, Martinoia E (2009). The *Arabidopsis* ATP-binding cassette protein *AtMRP5/AtABCC5* is a high affinity inositol hexakisphosphate transporter involved in guard cell signaling and phytate storage. J Biol Chem.

[14] Badone FC, Cassani E, Landoni M, Doria E, Panzeri D, Lago C, Mesiti F, Nielsen E, Pilu R (2010). The low phytic acid1-241 (lpa1-241) maize mutation alters the accumulation of anthocyanin pigment in the kernel. Planta.

[15] Tagashira Y, Shimizu T, Miyamoto M, Nishida S, Yoshida KT (2015). Overexpression of a gene involved in phytic acid biosynthesis substantially increases phytic acid and total phosphorus in rice seeds. Plants.

[16] Park J, Song W-Y, Ko D, Eom Y, Hansen TH, Schiller M, Lee TG, Martinoia E, Lee Y (2012). The phytochelatin transporters *ATABCC1* and *ATABCC2* mediate tolerance to cadmium and mercury. Plant J.

[17] Kato T, Tabata S, Sato S (2009). Analyses of expression and phenotypes of knockout lines for *Arabidopsis* ABCF subfamily members. Plant Biotech.

[18] Bessire M, Borel S, Fabre G, Carraca L, Efremova N, Yephremov A, Cao Y, Jetter R, Jacquat AC, Metraux JP (2011). A member of the pleiotropic drug resistance family of ATP binding cassette transporters is required for the formation of a functional cuticle in *Arabidopsis*. Plant Cell.

[19] Chen G, Komatsuda T, Ma JF, Nawrath C, Pourkheirandish M, Tagiri A, Hu Y-G, Sameri M, Li X, Zhao X (2011). An ATP-binding cassette subfamily G full transporter is essential for the retention of leaf water in both wild barley and rice. Proc Natl Acad Sci U S A.

[20] Fu S, Lu Y, Zhang X, Yang G, Chao D, Wang Z, Shi M, Chen J, Chao D-Y, Li R (2019). The ABC transporter *ABCG36* is required for cadmium tolerance in rice. J Exp Bot.

[21] Kim D-Y, Bovet L, Maeshima M, Martinoia E, Lee Y (2007). The ABC transporter *ATPDR8* is a cadmium extrusion pump conferring heavy metal resistance. Plant J.

[22] Bovet L, Eggmann T, Meylan-bettex M, Polier J, Kammer P, Marin E, Feller U, Martinoia E (2003). Transcript levels of *ATMRPS* after cadmium treatment: induction of *ATMRP3*. Plant Cell Environ.

[23] Emamverdian A, Ding Y, Mokhberdoran F, Xie Y (2015). Heavy metal stress and some mechanisms of plant defense response. Sci World J.

[24] Salla V, Hardaway CJ, Sneddon J (2011). Preliminary investigation of *Spartina alterniflora* for phytoextraction of selected heavy metals in soils from Southwest Louisiana. Microchem J.

[25] Yamaji N, Xia J, Mitani-Ueno N, Yokosho K, Feng Ma J (2013). Preferential delivery of zinc to developing tissues in rice is mediated by P-type heavy metal ATPase *OsHMA2*. Plant Physiol.

[26] Huang X-Y, Deng F, Yamaji N, Pinson SRM, Fujii-Kashino M, Danku J, Douglas A, Guerinot ML, Salt DE, Ma JF (2016). A heavy metal P-type ATPase *OsHMA4* prevents copper accumulation in rice grain. Nat Commun.

[27] Mikkelsen MD, Pedas P, Schiller M, Vincze E, Mills RF, Borg S, Møller A, Schjoerring JK, Williams LE, Baekgaard L, et al. Barley *HvHMA1* is a heavy metal pump involved in mobilizing organellar Zn and Cu and plays a role in metal loading into grains. PLoS One. 2012;7(11):–e49027. 10.1371/journal.pone.0049027 PMC3498361 23155447

[28] Tan J, Wang J, Chai T, Zhang Y, Feng S, Li Y, Zhao H, Liu H, Chai X (2013). Functional analyses of *TaHMA2*, a P_1B_-type ATPase in wheat. Plant Biotechnol J.

[29] Morel M, Crouzet J, Gravot A, Auroy P, Leonhardt N, Vavasseur A, Richaud P (2009). *AtHMA3*, a P_1B_-ATPase allowing cd/Zn/co/Pb vacuolar storage in *Arabidopsis*. Plant Physiol.

[30] Wang Z, Hobson N, Galindo L, Zhu S, Shi D, McDill J, Yang L, Hawkins S, Neutelings G, Datla R (2012). The genome of flax (*linum usitatissimum*) assembled de novo from short shotgun sequence reads. Plant J.

[31] You FM, Xiao J, Li P, Yao Z, Jia G, He L, Zhu T, Luo M-C, Wang X, Deyholos MK (2018). Chromosome-scale pseudomolecules refined by optical, physical and genetic maps in flax. Plant J.

[32] Jasinski M, Ducos E, Martinoia E, Boutry M (2003). The ATP-binding cassette transporters: structure, function, and gene family comparison between rice and *Arabidopsis*. Plant Physiol.

[33] Pang K, Li Y, Liu M, Meng Z, Yu Y (2013). Inventory and general analysis of the ATP-binding cassette (*ABC*) gene superfamily in maize (*Zea mays* L.). Gene.

[34] Cao Y, Zhao X, Liu Y, Wang Y, Wu W, Jiang Y, Liao C, Xu X, Gao S, Shen Y (2019). Genome-wide identification of ZmHMAs and association of natural variation in *ZmHMA2* and *ZmHMA3* with leaf cadmium accumulation in maize. PeerJ.

[35] Li N, Xiao H, Sun J, Wang S, Wang J, Chang P, Zhou X, Lei B, Lu K, Luo F (2018). Genome-wide analysis and expression profiling of the HMA gene family in *Brassica napus* under cd stress. Plant Soil.

[36] Bhati KK, Sharma S, Aggarwal S, Kaur M, Shukla V, Kaur J, Mantri S, Pandey AK (2015). Genome-wide identification and expression characterization of ABCC-MRP transporters in hexaploid wheat. Front Plant Sci.

[37] Çakır B, Kılıçkaya O (2013). Whole-genome survey of the putative ATP-binding cassette transporter family genes in *Vitis Vinifera*. PLoS One.

[38] Lane TS, Rempe CS, Davitt J, Staton ME, Peng Y, Soltis DE, Melkonian M, Deyholos M, Leebens-Mack JH, Chase M (2016). Diversity of ABC transporter genes across the plant kingdom and their potential utility in biotechnology. BMC Biotechnol.

[39] Shivaraj SM, Deshmukh RK, Rai R, Bélanger R, Agrawal PK, Dash PK (2017). Genome-wide identification, characterization, and expression profile of aquaporin gene family in flax (*Linum usitatissimum*). Sci Rep.

[40] Corbin C, Drouet S, Markulin L, Auguin D, Lainé É, Davin LB, Cort JR, Lewis NG, Hano C (2018). A genome-wide analysis of the flax (*Linum usitatissimum* L.) dirigent protein family: from gene identification and evolution to differential regulation. Plant Mol Biol.

[41] Eom SH, Hyun TK (2016). Genome-wide identification and transcriptional expression analysis of chalcone synthase in flax (*Linum usitatissimum* L.). Gene Rep.

[42] Ali E, Saand MA, Khan AR, Shah JM, Feng S, Ming C, Sun P. Genome-wide identification and expression analysis of detoxification efflux carriers (DTX) genes family under abiotic stresses in flax. Physiol Plant. 2020. 10.1111/ppl.13105. 10.1111/ppl.13105 32270877

[43] Barvkar VT, Pardeshi VC, Kale SM, Kadoo NY, Gupta VS (2012). Phylogenomic analysis of UDP glycosyltransferase 1 multigene family in *Linum usitatissimum* identified genes with varied expression patterns. BMC Genomics.

[44] Fan W, Liu C, Cao B, Qin M, Long D, Xiang Z, Zhao A (2018). Genome-wide identification and characterization of four gene families putatively involved in cadmium uptake, translocation and sequestration in mulberry. Front Plant Sci.

[45] Verret F, Gravot A, Auroy P, Leonhardt N, David P, Nussaume L, Vavasseur A, Richaud P (2004). Overexpression of *AtHMA4* enhances root-to-shoot translocation of zinc and cadmium and plant metal tolerance. FEBS Lett.

[46] Takahashi R, Ishimaru Y, Shimo H, Ogo Y, Senoura T, Nishizawa NK, Nakanishi H (2012). The *OsHMA2* transporter is involved in root-to-shoot translocation of Zn and cd in rice. Plant Cell Environ.

[47] Kuromori T, Miyaji T, Yabuuchi H, Shimizu H, Sugimoto E, Kamiya A, Moriyama Y, Shinozaki K (2010). ABC transporter *AtABCG25* is involved in abscisic acid transport and responses. Proc Natl Acad Sci U S A.

[48] Song W-Y, Yamaki T, Yamaji N, Ko D, Jung K-H, Fujii-Kashino M, An G, Martinoia E, Lee Y, Ma JF (2014). A rice ABC transporter, *OsABCC1*, reduces arsenic accumulation in the grain. Proc Natl Acad Sci U S A.

[49] Kim D-Y, Jin J-Y, Alejandro S, Martinoia E, Lee Y (2010). Overexpression of *AtABCG36* improves drought and salt stress resistance in *Arabidopsis*. Physiol Plant.

[50] Takeda S, Iwasaki A, Tatematsu K, Okada K (2014). The half-size abc transporter folded petals 2/ABCG13 is involved in petal elongation through narrow spaces in *Arabidopsis thaliana* floral buds. Plants.

[51] Acuña-Galindo MA, Mason RE, Subramanian NK, Hays DB (2015). Meta-analysis of wheat QTL regions associated with adaptation to drought and heat stress. Crop Sci.

[52] Gu X, Zou Y, Su Z, Huang W, Zhou Z, Arendsee Z, Zeng Y (2013). An update of diverge software for functional divergence analysis of protein family. Mol Biol Evol.

[53] Jiao Y, Wickett NJ, Ayyampalayam S, Chanderbali AS, Landherr L, Ralph PE, Tomsho LP, Hu Y, Liang H, Soltis PS (2011). Ancestral polyploidy in seed plants and angiosperms. Nature.

[54] Clark JW, Donoghue PCJ (2018). Whole-genome duplication and plant macroevolution. Trends Plant Sci.

[55] Segraves KA (2017). The effects of genome duplications in a community context. New Phytol.

[56] Moghe GD, Shiu SH (2014). The causes and molecular consequences of polyploidy in flowering plants. Ann N Y Acad Sci.

[57] Cannon SB, Mitra A, Baumgarten A, Young ND, May G (2004). The roles of segmental and tandem gene duplication in the evolution of large gene families in *Arabidopsis thaliana*. BMC Plant Biol.

[58] Khan N, Fatima F, Haider MS, Shazadee H, Liu Z, Zheng T, Fang J (2019). Genome-wide identification and expression profiling of the polygalacturonase (PG) and pectin methylesterase (PME) genes in grapevine (*Vitis vinifera* L.). Int J Mol Sci.

[59] Shazadee H, Khan N, Wang J, Wang C, Zeng J, Huang Z, Wang X (2019). Identification and expression profiling of protein phosphatases (*PP2C*) gene family in *Gossypium hirsutum* L. Int J Mol Sci.

[60] Die JV, Gil J, Millan T (2018). Genome-wide identification of the auxin response factor gene family in *Cicer arietinum*. BMC Genomics.

[61] Juretic N, Hoen DR, Huynh ML, Harrison PM, Bureau TE (2005). The evolutionary fate of mule-mediated duplications of host gene fragments in rice. Genome Res.

[62] Lynch M, Conery JS (2000). The evolutionary fate and consequences of duplicate genes. Science.

[63] Li J, Zhang Z, Vang S, Yu J, Wong GK, Wang J (2009). Correlation between *ka/ks* and *ks* is related to substitution model and evolutionary lineage. J Mol Evol.

[64] Mercereau-Puijalon O, Barale JC, Bischoff E (2002). Three multigene families in *plasmodium* parasites: facts and questions. Int J Parasitol.

[65] Verrier PJ, Bird D, Burla B, Dassa E, Forestier C, Geisler M, Klein M, Kolukisaoglu Ü, Lee Y, Martinoia E (2008). Plant ABC proteins – a unified nomenclature and updated inventory. Trends Plant Sci.

[66] Finn RD, Clements J, Eddy SR (2011). HMMER web server: interactive sequence similarity searching. Nucleic Acids Res.

[67] El-Gebali S, Mistry J, Bateman A, Eddy SR, Luciani A, Potter SC, Qureshi M, Richardson LJ, Salazar GA, Smart A (2018). The Pfam protein families database in 2019. Nucleic Acids Res.

[68] Goodstein DM, Shu S, Howson R, Neupane R, Hayes RD, Fazo J, Mitros T, Dirks W, Hellsten U, Putnam N (2012). Phytozome: a comparative platform for green plant genomics. Nucleic Acids Res.

[69] Marchler-Bauer A, Bo Y, Han L, He J, Lanczycki CJ, Lu S, Chitsaz F, Derbyshire MK, Geer RC, Gonzales NR (2017). CDD/SPARCLE: functional classification of proteins via subfamily domain architectures. Nucleic Acids Res.

[70] Letunic I, Bork P (2017). 20 years of the smart protein domain annotation resource. Nucleic Acids Res.

[71] Kumar S, Stecher G, Tamura K (2016). MEGA7: molecular evolutionary genetics analysis version 7.0 for bigger datasets. Mol Biol Evol.

[72] Wang Y, Tang H, Debarry JD, Tan X, Li J, Wang X, Lee T-H, Jin H, Marler B, Guo H (2012). MCScanX: a toolkit for detection and evolutionary analysis of gene synteny and collinearity. Nucleic Acids Res.

[73] Koch MA, Haubold B, Mitchell-Olds T (2000). Comparative evolutionary analysis of chalcone synthase and alcohol dehydrogenase loci in *Arabidopsis*, *Arabis*, and related genera (*Brassicaceae*). Mol Bio Evol.

[74] Bailey TL, Boden M, Buske FA, Frith M, Grant CE, Clementi L, Ren J, Li WW, Noble WS. MEME suite: tools for motif discovery and searching. Nucleic Acids Res. 2009;37(Web Server issue):W202-208. 10.1093/nar/gkp335 PMC2703892 19458158

[75] Chen C, Chen H, Zhang Y, Thomas HR, Frank MH, He Y, Xia R (2020). TBtools: an integrative toolkit developed for interactive analyses of big biological data. Mol Plant.

[76] Horton P, Park K-J, Obayashi T, Fujita N, Harada H, Adams-Collier CJ, Nakai K (2007). WoLF PSORT: protein localization predictor. Nucleic Acids Res.

[77] Savojardo C, Martelli Pier L, Fariselli P, Profiti G, Casadio R (2018). BUSCA: an integrative web server to predict subcellular localization of proteins. Nucleic Acids Res.

[78] Yu Y, Ouyang Y, Yao W (2017). shinyCircos: an R/shiny application for interactive creation of circos plot. Bioinformatics.

[79] Szklarczyk D, Gable AL, Lyon D, Junge A, Wyder S, Huerta-Cepas J, Simonovic M, Doncheva NT, Morris JH, Bork P (2018). STRING v11: protein–protein association networks with increased coverage, supporting functional discovery in genome-wide experimental datasets. Nucleic Acids Res.

[80] Shannon P, Markiel A, Ozier O, Baliga NS, Wang JT, Ramage D, Amin N, Schwikowski B, Ideker T (2003). Cytoscape: a software environment for integrated models of biomolecular interaction networks. Genome Res.

[81] Venglat P, Xiang D, Qiu S, Stone SL, Tibiche C, Cram D, Alting-Mees M, Nowak J, Cloutier S, Deyholos M (2011). Gene expression analysis of flax seed development. BMC Plant Biol.

[82] Bolger AM, Lohse M, Usadel B (2014). Trimmomatic: a flexible trimmer for illumina sequence data. Bioinformatics.

[83] Bray NL, Pimentel H, Melsted P, Pachter L (2016). Near-optimal probabilistic RNA-Seq quantification. Nat Biotechnol.

[84] Robinson MD, McCarthy DJ, Smyth GK (2010). edgeR: a bioconductor package for differential expression analysis of digital gene expression data. Bioinformatics.

[85] Metsalu T, Vilo J (2015). ClustVis: a web tool for visualizing clustering of multivariate data using principal component analysis and heatmap. Nucleic Acids Res.

