# Supplementary material for: Genome-wide identification of ATP binding cassette (ABC) transporter and heavy metal associated (HMA) gene families in flax (Linum usitatissimum L.)
Source: BMC Genomics. 2020 Oct 19;21:722. doi: 10.1186/s12864-020-07121-9 (PMC7574471; doi:10.1186/s12864-020-07121-9)
Supplement: Supplementary file 1 — Additional file 1: Figure S1. Multidimensional scaling (MDS) plot of nine different tissues displaying the relative similarities between the biological replicates based on the log fold change values. Figure S2. Interaction network of ABC transporter genes (a) and HMA genes (b). Figure S3. Schematic representations of the two most stable genes among nine Cd candidate genes based on their conserved gene structure (a), gene expression (b), non-synonymous/synonymous substitution rates (c), and Pearson correlation coefficient (PCC) (d). [file 12864_2020_7121_MOESM1_ESM.docx]

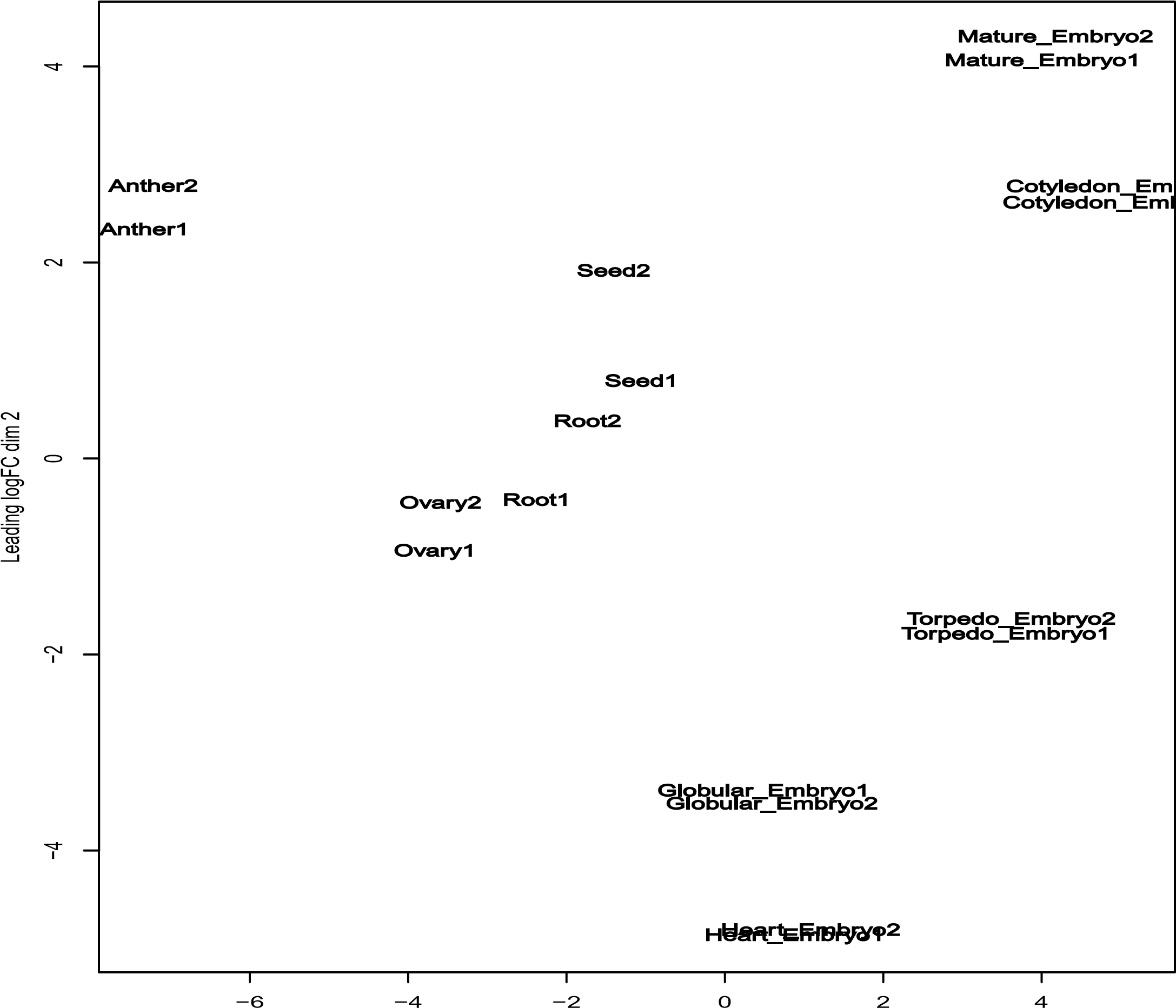


**Figure S1.** Multidimensional scaling (MDS) plot of nine different organs displaying the relative similarities between the biological replicates based on the log fold change values.


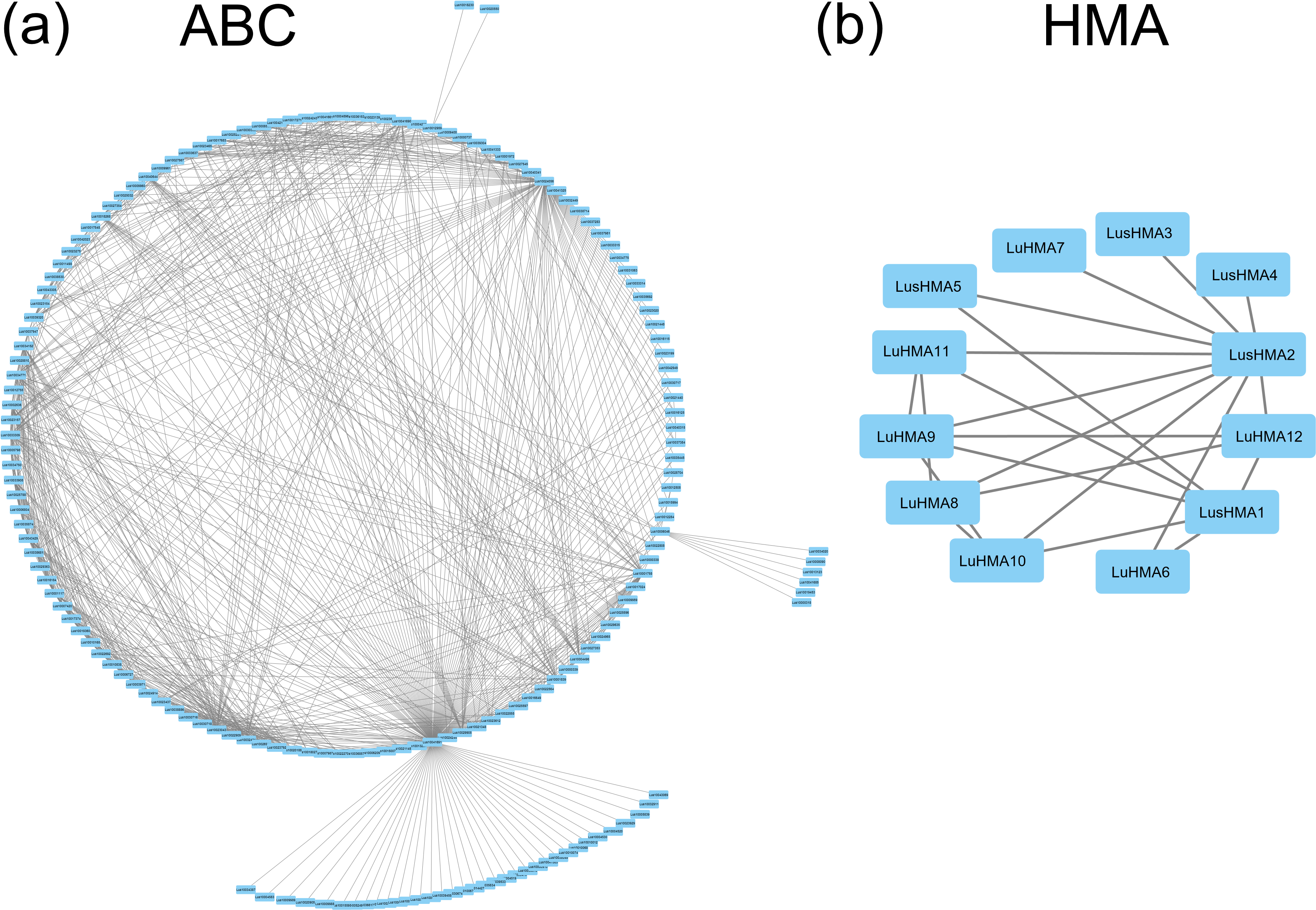


**Figure S2.** Interaction network of ABC transporter genes (a) and HMA genes (b).


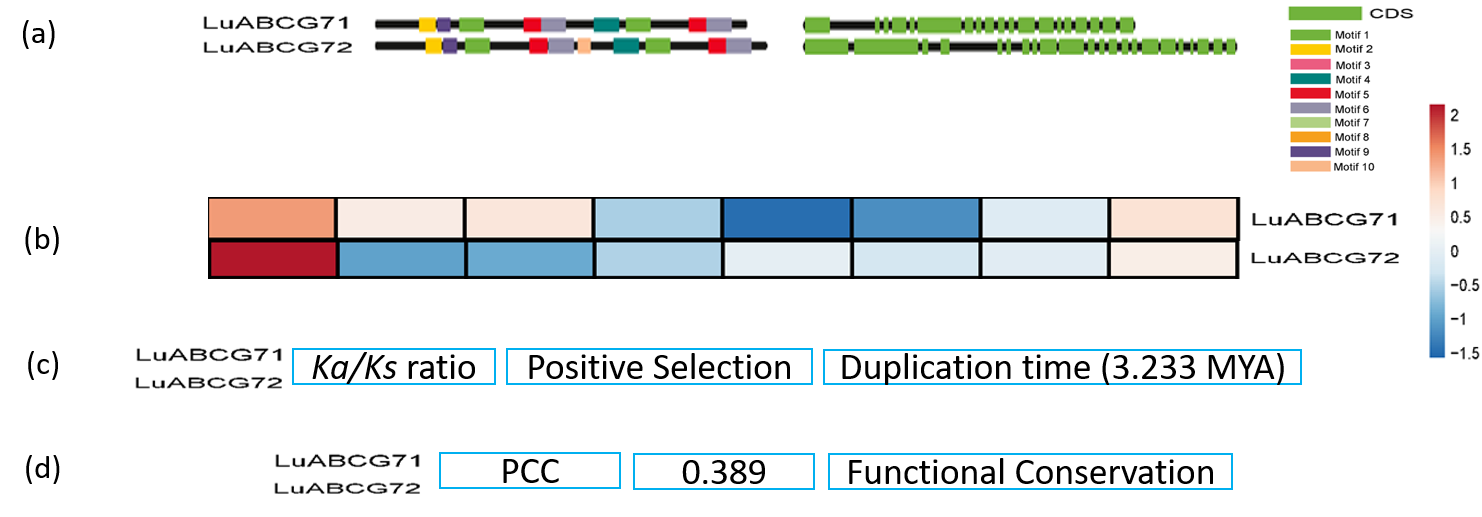


**Figure S3.** Schematic representations of the two most stable genes among nine Cd candidate genes based on their conserved gene structure (a), gene expression (b), non-synonymous/synonymous substitution rates (c), and Pearson correlation coefficient (PCC) (d).
